# Diagnostic value of multi-tumor-associated autoantibody expression in esophageal squamous cell carcinoma and correlation of clinical features

**DOI:** 10.3389/fimmu.2024.1518431

**Published:** 2024-12-18

**Authors:** Sihao Zhou, Kejun Liu, Yuyu Yang, Chuan Yuan, Yi Liu, Yi Liang, Wenhao Li, Jingjing Zhang, Hongyu Ye, Sheng Gong, Yingmeng Wu, Weizhao Huang

**Affiliations:** ^1^ Zhongshan City People’s Hospital, Zhongshan, Guangdong, China; ^2^ The Second Affiliated Hospital of Chengdu Medicial College, China National Nuclear Corporation 416 Hospital, Chengdu, Sichuan, China; ^3^ Guangdong Medical University, Zhangjiang, Guangdong, China

**Keywords:** esophageal squamous cell carcinoma, 7-tumor associated autoantibodies, combination test, diagnostic value, clinical features

## Abstract

**Objective:**

This study aimed to investigate the diagnostic value of 7-tumor associated autoantibodies (7-TAAB) and to evaluate the relationship between 7-TAAB and clinical features in esophageal squamous cell carcinoma (ESCC), which can be used to guide clinical diagnosis and treatment and achieve its clinical value.

**Methods:**

(1) Blood specimens were collected from patients with ESCC who had not previously received antitumor therapy (ESCC group) and those who had normal medical check-ups in the hospital during the same period (control group). The concentrations of 7-TAAB (P53, PGP9.5, SOX2, GAGE7, GBU4-5, MAGE A1, and CAGE) in serum were determined by enzyme-linked immunosorbent assay. The concentrations of 7-TAAB were compared between the ESCC and control groups, and the positive rate of 7-TAAB was calculated to determine the sensitivity, specificity, and accuracy of 7-TAAB. The diagnostic value of 7-TAAB was analyzed using the receiver operating characteristic (ROC) curve. (2) The clinical data of patients with ESCC were collected and the correlation between the rate of 7-TAAB and clinical features was analyzed.

**Results:**

(1) The serum levels and positivity rates of five antibodies (PGP9.5, SOX2, GBU4-5, MAGE-A1, and CAGE) were higher in the ESCC group than in the control group (*P* < 0.05) and the positive expression rate of the combined serum 7-TAAB in the ESCC group was significantly higher than that in the control group (*P* < 0.05). (2) The sensitivity of single antibody detection was 4.20%–17.65%, with a specificity of 96.49%–100%, and accuracy of 51.07%–57.94%. The sensitivity of 7-TAAB combined detection was 49.58%, the specificity was 92.98%, and the accuracy was 70.81%. (3) The ROC curve showed that the 7-TAAB combined test had a certain diagnostic value for ESCC and that its diagnostic efficacy was significantly higher than that of the single autoantibody tests. The diagnostic efficacy of the combined test with the remaining five antibodies (PGP9.5, SOX2, GBU4-5, MAGE-A1, and CAGE) was similar to that of the 7-TAAB combined test after eliminating the two antibodies with low expression rates. (4) Univariate analysis revealed significant differences in the positive expression rates of the 7-TAAB combination test in terms of age, hemoglobin level, albumin level, tumor location, tumor length, lymph node stage, and tumor clinical stage (*P* < 0.05), and multivariate analysis revealed that age and lymph node stage were independent factors affecting antibody expression.

**Conclusion:**

The multi-tumor-associated autoantibody combination test not only has a good auxiliary diagnostic value but also closely correlates with the clinical features of ESCC.

## 1 Introduction

According to the global oncology data released by the International Agency for Research on Cancer in 2022, esophageal cancer ranks 11th and 7th in terms of new cases and deaths, respectively. China is a particularly high-risk area for esophageal cancer, of which 95.5% of esophageal squamous cell carcinoma (ESCC), making it a major country for ESCC worldwide (1, 2). In recent years, studies have shown that in the early stages of tumor development, abnormally expressed tumor proteins can be recognized by the body’s immune system, which then stimulates autoimmune responses to produce numerous autoantibodies, which are referred to as tumor-associated autoantibodies (TAAB) (3). The 7-TAAB, which consist of the P53, PGP9.5, SOX2, GAGE7, GBU4-5, MAGE A1, and CAGE antibodies, represent an antibody spectrum. As a new serum marker, the 7-TAAB detection method, which has been applied in clinical tumor research (4, 5), has several advantages, including that it is convenient, inflicts less trauma on patients than other methods, and is easy to obtain specimens for. P53 is one of the earliest discovered oncogenes that participates in the development of tumors through DNA repair and apoptosis-inducing cells (6). PGP9.5, a ubiquitin hydroxyl-terminal hydrolase, plays a key role in tumor development by increasing the deubiquitination of cell cycle proteins (7). SOX2 is an important transcription factor that regulates cell development and is closely associated with various tumors (8). GBU4-5, an RNA-conjugating enzyme that regulates processes such as cell growth and division by encoding the DEAD-box protein to achieve oncogenesis. GAGE7, MAGE A1, and CAGE, as members of the tumor-testis antigen family, accelerate tumor formation, delay tumor cell apoptosis, and promote tumor cell metastasis (9–11). At present, the 7-TAAB detection method has not been applied in esophageal cancer research. Therefore, this study aimed to investigate the diagnostic value of 7-TAAB and evaluate the relationship between 7-TAAB and clinical features in ESCC, which can be used to guide clinical diagnosis and treatment and achieve good clinical value.

## 2 Patients and methods

### 2.1 Patients

One hundred and nineteen patients with ESCC confirmed by histopathology who visited our hospital between April 2022 and November 2023 were selected as the ESCC group, and 114 healthy people with a normal physical examination in the same period were selected as the control group. Tumor staging was based on the 8th edition of the International TNM staging criteria for esophageal and gastric junction cancer (AJCC/UICC). This study was approved by the Ethics Committee of Zhongshan City People’s Hospital (Zhongshan, China) under approval number 2024-074. This study was also conducted in accordance with the Declaration of Helsinki. All participants provided their written informed consent prior to their inclusion in the study.

The inclusion criteria were as follows: (1) all patients in the experimental group were diagnosed with ESCC by pathology and were not treated with any antitumor therapy before admission; (2) all patients had normal liver and kidney functions; and (3) all patients had complete data.

The exclusion criteria were as follows: (1) previous or current malignant tumors in other parts of the body; (2) esophageal metastases from other malignant tumors; and (3) combined autoimmune diseases such as type I diabetes mellitus, rheumatoid arthritis, or diseases that affect immune function (e.g., HIV).

### 2.2 Methods

In both groups, 5 ml of peripheral venous blood samples were collected in the early morning fasting state, and the serum was separated by centrifugation at a radius of 10 cm and a rotational speed of 4000 rpm for 10 min, before being subjected to enzyme-linked immunosorbent assay (ELISA). The 7-TAAB were detected using an ELISA kit produced by Hangzhou Kaipaul Bio-technology Co., with the following positive reference values: P53 ≥ 13.1 U/mL, PGP9.5 ≥ 11.1 U/mL, SOX2 ≥ 10.3 U/mL, GAGE7 ≥ 14.4 U/mL, GBU4.5 ≥ 7.0 U/mL, MAGE A1 ≥ 11.9 U/mL, and CAGE ≥ 7.2 U/mL. Interpretation of the results: If the concentration of any of the antibodies is higher than the positive value, the test is considered “positive”.

### 2.3 Statistical analysis

All data were analyzed using the SPSS software(version 26.0). For measures that conformed to a normal distribution, the mean ± standard deviation (x ± s) was expressed using the independent samples *t*-test for measures that did not conform to a normal distribution, the median (lower quartile, upper quartile) [M(P25, P75)] was expressed, and the two groups were compared using the Mann–Whitney U test. Count data were expressed as cases or percentages (%), and comparisons between the two groups were made using the chi-squared or Fisher’s exact probability method on a four-compartment scale. Comparisons between multiple groups were made using the chi-square test on the RxC list or Fisher’s exact probability method on the RxC list. Diagnostic efficacy was evaluated using receiver operating characteristic curve (ROC curve). For multivariate analysis, binary logistic regression analysis was used (*P* ≤ 0.05 was considered a statistically significant difference).

## 3 Results

### 3.1 Baseline characteristics of patients

A total of 233 study subjects were included, comprising 119 patients in the ESCC group and 114 patients in the control group, with no significant difference in sex, age, or history of tobacco and alcohol addiction between the two groups (P > 0.05) (see **Table 1** for details).

**Table 1:** Baseline characteristics of patients.

	ESCC group n = 119	Control group n = 114	t-value	*χ^2^* value	*P*-value
Age	61.16 ± 8.48	60.07 ± 9.05	0.948		0.344
Sex				0.323	0.570
Male	110 (92.4%)	103 (90.4%)			
Female	9 (7.6%)	11 (9.6%)			
Smoke cigarettes				0.002	0.969
Yes	78 (65.5%)	75 (65.8%)			
No	41 (34.5%)	39 (34.2%)			
Drink wine				0.467	0.494
Yes	70 (58.8%)	62 (54.4%)			
No	49 (41.2%)	52 (45.6%)			
Diabetes				0.720	0.396
Yes	34 (28.6%)	27 (23.7%)			
No	85 (61.4%)	87 (76.3%)			
Hypertensive				0.030	0.862
Yes	42 (35.3%)	39 (34.2%)			
No	77 (64.7%)	75 (65.8%)			
Coronary heart disease				1.570	0.210
Yes	30 (25.2%)	21 (18.4%)			
No	89 (74.8%)	93 (81.6%)			

### 3.2 Comparison of serum 7-TAAB levels and positive expression rates between the two groups

**Figure 1:**
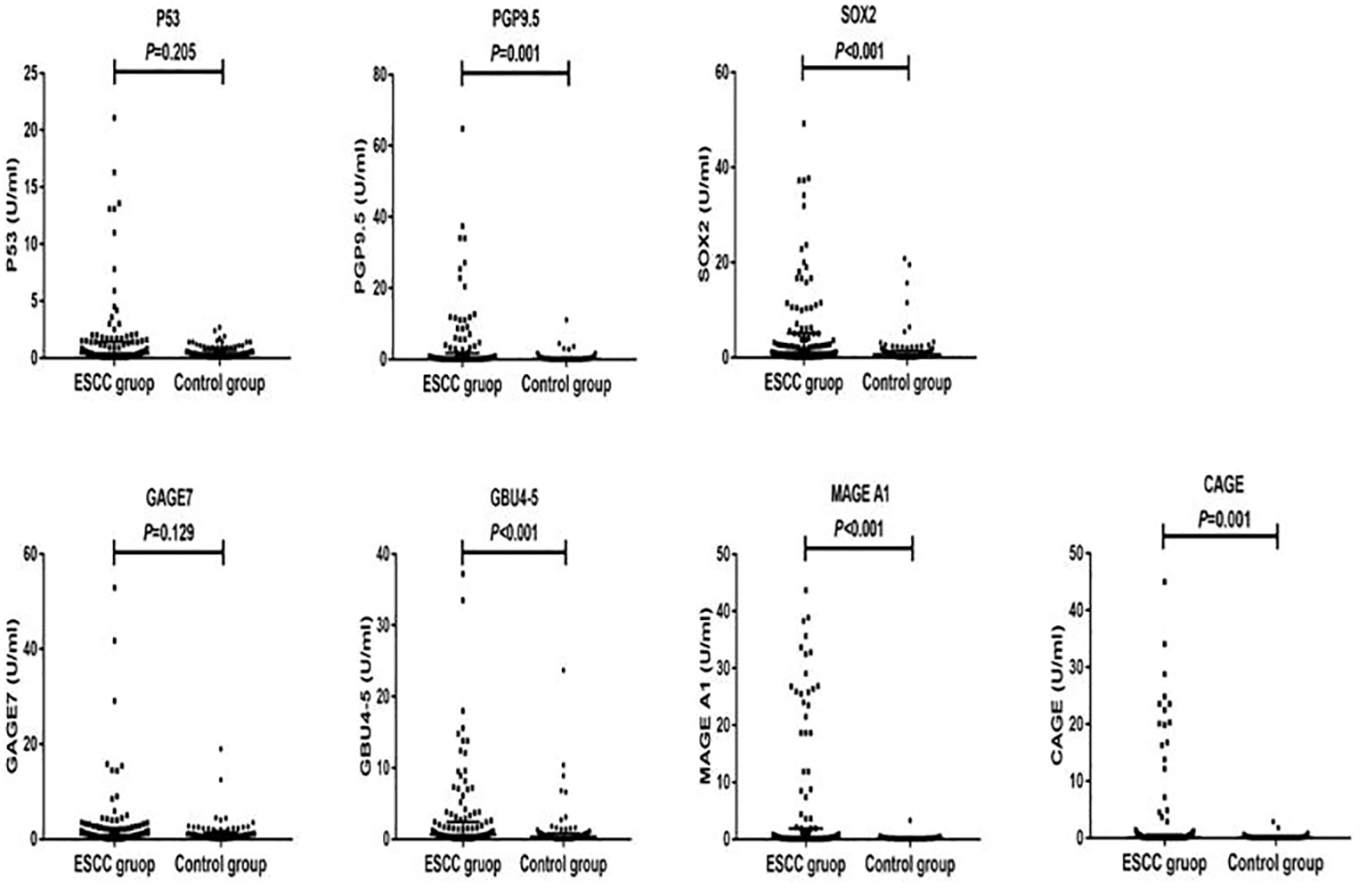
Distribution of serum expression levels of 7-TAAB in the two groups. **Figure 1** shows the distribution of P53, PGP9.5, SOX2, GAGE7, GBU4-5, MAGE A1, and CAGE serum levels in the ESCC and control groups.

The antibody levels and positive expression rates of PGP9.5, SOX2, GBU4-5, MAGE A1, and CAGE were significantly higher in the ESCC group than in the control group (*P* < 0.05). The positive expression rate of the serum 7-TAAB combination test in patients in the ESCC group was significantly higher than that in the control group (*P* < 0.05) (for details, see **Tables 2**, **3**; **Figure 1**).

**Table 2:** Comparison of 7-TAAB serum expression levels between the two groups.

TAAB	ESCC group n = 119	Normal group n = 114	Z-value	*P*-value
P53	0.40 (0.10, 1.40)	0.40 (0.10, 0.70)	1.267	0.205
PGP9.5	0.20 (0.10, 1.70)	0.10 (0.10, 0.30)	3.260	0.001^▴^
SOX2	1.10 (0.20, 5.00)	0.70 (0.10, 1.40)	3.550	<0.001^▴^
GAGE7	0.90 (0.40, 2.10)	0.80 (0.30, 1.40)	1.517	0.129
GBU4-5	0.60 (0.30, 2.40)	0.35 (0.10, 0.80)	3.593	<0.001^▴^
MAGE A1	0.20 (0.10, 1.90)	0.10 (0.10, 0.20)	4.245	<0.001^▴^
CAGE	0.12 (0.10, 0.60)	0.10 (0.10, 0.13)	3.436	0.001^▴^

^▴^: *P*<0.05 the median (lower quartile, upper quartile) [M(P25, P75)].

**Table 3:** Comparison of 7-TAAB seropositivity rates between the two groups.

TAAB	ESCC group n = 119	Normal group n = 114	*χ^2^* value	*P*-value
P53	5 (4.20%)	0	3.099	0.078
PGP9.5	13 (10.92%)	1 (0.88%)	10.407	0.001^▴^
SOX2	21 (17.65%)	4 (3.51%)	12.151	<0.001^▴^
GAGE7	6 (5.04%)	1 (0.88%)	2.184	0.139
GBU4-5	16 (13.45%)	2 (1.75%)	11.163	0.001^▴^
MAGE A1	21 (17.65%)	0	22.110	<0.001^▴^
CAGE	14 (11.76%)	0	14.269	<0.001^▴^
7-TAAB	59 (49.58%)	8 (7.02%)	51.484	<0.001^▴^

^▴^: *P*<0.05.

### 3.3 Diagnostic value of 7-TAAB single and combined tests for ESCC

**Figure 2:**
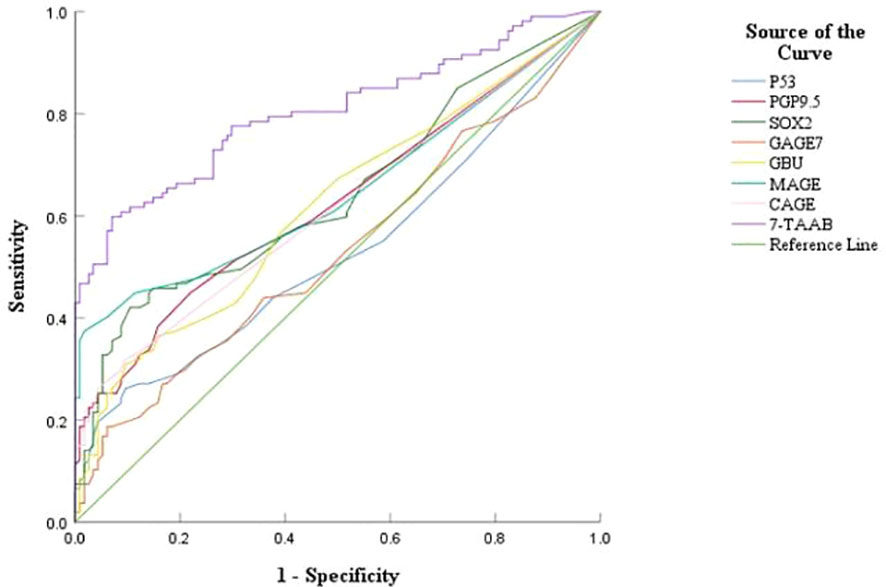
ROC curves of 7-TAAB single and combined tests for the diagnosis of ESCC. **Figure 2** compares the ROC curves for the combined P53, PGP9.5, SOX2, GAGE7, GBU4-5, MAGE A1, CAGE, and 7-TAAB assays.

**Figure 3:**
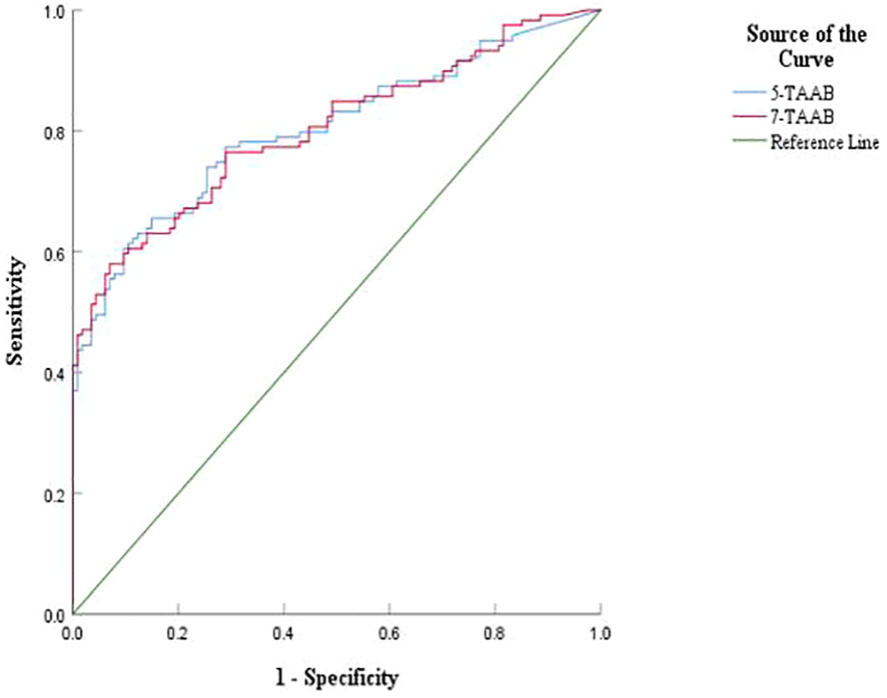
Comparison of ROC curves between 5-TAAB and 7-TAAB co-tests for ESCC diagnosis. 5-TAAB refers to the five tumor-associated autoantibodies SOX2, PGP9.5, GBU4-5, MAGE-A1, and GAGE.

The sensitivity, specificity, and accuracy of the single antibody test were 4.20%–17.65%, 96.49%–100%, and 51.07%–57.94% respectively. The ROC curve showed that except for P53 (AUC = 0.547, *P* = 0.211) and GAGE7 (AUC = 0.557, *P* = 0.130), the remaining five antibodies played a role in the diagnosis of ESCC, and the diagnostic value of MAGE A1 was the highest (AUC = 0.653, *P* < 0.001). The sensitivity, specificity, and accuracy of the 7-TAAB combination test were 49.58%, 92.98%, and 70.81% respectively, and the AUC value of the 7-TAAB combination test was 0.802 (*P* < 0.001). Therefore, the 7-TAAB combination test not only has diagnostic value for ESCC, but it is also significantly better than the individual antibody tests. By eliminating the P53 and GAGE7 antibodies, the composition of the 5-TAAB combination test and the diagnostic value of the 5-TAAB combined test and the 7-TAAB combined test were the same (for details, see **Table 4**; **Figures 2**, **3**).

**Table 4:** Diagnostic value of 7-TAAB single and combined tests for ESSC in the two groups.

TAAB	ESCC group n = 119	Control group n = 114	Sensitivity (%)	Specificity (%)	Accuracy (%)	Youden index	PPV (%)	NPV (%)	AUC	*P*-value
P53	5	0	4.20%	100%	51.07%	0.04	100.00%	50.00%	0.547	0.211
PGP9.5	13	1	10.92%	99.12%	54.08%	0.10	92.86%	54.85%	0.616	0.002^▴^
SOX2	21	4	17.65%	96.49%	56.22%	0.14	84.00%	52.88%	0.634	<0.001^▴^
GAGE7	6	1	5.04%	99.12%	51.07%	0.04	85.71%	50.00%	0.557	0.130
GBU4-5	16	2	13.45%	98.25%	54.94%	0.12	88.89%	52.09%	0.635	<0.001^▴^
MAGE A1	21	0	17.65%	100.00%	57.94%	0.18	100.00%	53.77%	0.653	<0.001^▴^
CAGE	14	0	11.76%	100.00%	54.94%	0.12	100.00%	54.55%	0.609	0.004^▴^
7-TAAB	59	8	49.58%	92.98%	70.81%	0.43	88.06%	63.86%	0.802	<0.001^▴^
5-TAAB	55	7	46.22%	93.86%	69.53%	0.40	88.71%	62.57%	0.802	<0.001^▴^

5-TAAB refers to the five tumor-associated autoantibodies SOX2, PGP9.5, GBU4-5, MAGE-A1, and GAGE, PPV: Positive predictive value, NPV: Negative predictive value; ^▴^: *P*<0.05.

### 3.4 Relationship between the clinical features of ESCC and the positive expression of 7-TAAB

The expression of 7-TAAB was significant with regard to age, hemoglobin level, albumin level, tumor location, tumor length, lymph node stage, and tumor clinical stage, whereas there was no significant difference between sex, body mass index, degree of differentiation, depth of infiltration, history of smoking and drinking, and distant metastasis (*P* > 0.05). Age and N stage were found to be independent factors influencing 7-TAAB expression in ESCC by multifactorial analysis (*P* > 0.05) (for details, see **Tables 5**, **6**).

**Table 5:** Relationship between 7-TAAB co-expression and clinicopathological features of ESCC.

Clinical features	7-TAAB positive (n = 59)	TAAB negatives (n = 60)	*χ^2^* value	*P*-value
Sex			0.518	0.472
Male	53 (89.8%)	57 (95%)		
Female	6 (10.2%)	3 (5%)		
Age (y)			6.222	0.013^▴^
< 60	20 (33.9%)	34 (56.7%)		
≥ 60	39 (66.1%)	26 (43.3%)		
BMI				
< 24	45 (76.3%)	44 (73.3%)	0.136	0.712
≥ 24	14 (23.7%)	16 (26.7%)		
Hb			8.241	0.004^▴^
< 130 g/L	32 (54.2%)	17 (28.3%)		
≥ 130 g/L	27 (45.8%)	43 (71.7%)		
ALB			4.626	0.031^▴^
< 40 g/L	40 (67.8%)	29 (48.3%)		
≥ 40 g/L	19 (32.2%)	31 (51.7%)		
Tumor location				0.039^▴^
Cervical and upper thoracic segments	7 (11.9%)	2 (3.3%)		
Middle thoracic segment	19 (32.2%)	12 (20.0%)		
Lower thoracic segment	33 (55.9%)	46 (76.7%)		
Tumor length			5.245	0.022^▴^
< 6 cm	24 (40.7%)	37 (61.7%)		
≥ 6 cm	35 (59.3%)	23 (38.3%)		
Degree of polarization			1.383	0.501
Lower	15 (25.4%)	17 (28.3%)		
Middle	35 (59.3%)	38 (63.3%)		
Higher	9 (15.3%)	5 (8.4%)		
T stage				
T1-2	5 (8.5%)	13 (21.7%)	4.097	0.141
T3	51 (86.4%)	45 (75.0%)		
T4	3 (5.1%)	2 (3.3%)		
N stage			12.987	0.005^▴^
N0	7 (11.9%)	12 (20.0%)		
N1	12 (20.3%)	23 (38.3%)		
N2	35 (59.3%)	16 (26.7%)		
N3	5 (8.5%)	9 (15.0%)		
M stage			0.117	0.773
M0	56 (94.9%)	55 (91.7%)		
M1	3 (5.1%)	5 (8.3%)		
CTNM			6.738	0.034^▴^
I-II	8 (13.6%)	17 (28.3%)		
III	43 (72.8%)	30 (50%)		
IV	8 (13.6%)	13 (21.7%)		

CTNM, clinical staging ^▴^: *P*<0.05.

**Table 6:** Multifactorial analysis of clinical characteristics of patients with ESCC with positive expression of the combined 7-TAAB assay.

variant	β	SE	Wald	P-value	OR	95% CI
Age	0.998	0.420	5.639	0.018^▴^	2.713	1.190–6.183
N stage (N1 vs. N0)	0.286	0.796	0.129	0.719	1.331	0.280–6.329
N stage (N2 vs. N0)	–0.089	0.707	0.016	0.900	0.915	0.229–3.655
N stage (N3 vs. N0)	1.364	0.671	4.133	0.042^▴^	3.913	1.050–14.581

^▴^: *P* < 0.05.

## 4 Discussion

Currently, there are no effective tumor markers for esophageal cancer. Although tumor-associated autoantibodies(TAAB) have been used in the study of esophageal squamous carcinoma, several studies have shown that their sensitivity is low at about 10%-30% and their accuracy is not high, so it is particularly important to find new antibody markers (12, 13). The 7-TAAB antibody spectrum has been approved by the China Food and Drug Administration in China. It has been widely used in the early diagnosis of lung cancer, with a sensitivity as high as 47%–65% and specificity as high as 51–81% (14–16), and its diagnostic accuracy has been increasingly recognized worldwide.

No studies have been found on the application of 7-TAAB to esophageal cancer, and only individual P53, GAGE7, and CAGE antibodies have been used in the clinical diagnosis of esophageal cancer (17–20). Our results revealed that the antibody levels and positive expression rates of PGP9.5, SOX2, GBU4-5, MAGE-A1, and CAGE were higher than those in the control group, whereas no statistical difference was found in the levels or positive expression rates of P53 and GAGE7, a finding that was contrary to that of Wang and Kunizaki (19, 21). The P53 and GAGE7 antibodies are the most common tumor antibodies, with several studies (19, 21, 22) showing a significant difference in expression between patients with esophageal cancer and healthy controls; however, the low expression levels of P53 and GAGE7 in the present study may be related to the interception of the reference values of antibody levels, individual differences between patients and the accuracy of different manufacturers of detection reagents. Therefore, we need to expand the sample size and adjust the reference values for antibody levels for further in-depth study.

The diagnostic efficacy of the 7-TAAB test for ESCC was evaluated by analyzing the sensitivity, specificity, and accuracy indices. Although the specificity of the single antibody test was 96.49%–100%, the accuracy was only 50%–60%, which was of low diagnostic value; the reason for this is that the sensitivities were all low, ranging from 4.20% to 17.65%, indicating that the single antibody does not yet meet the clinical requirements. By unifying the seven antibodies, we found that the accuracy reached 70.81%, the sensitivity was increased to 49.53%, and the specificity (92.98%) was also high, which was consistent with the conclusions of Sun and Zhang (17, 18), who used multiple antibodies to diagnose esophageal cancer and suggested that the combined detection of 7-TAAB has a certain clinical diagnostic value and can be used to distinguish ESCC in the population. Although the sensitivity of the 7-TAAB combination test is low, and it is clearly insufficient for early screening of ESCC, its specificity is high, and it can be used for auxiliary diagnosis of ESCC.

The results of Xiao et al. (20), showed that the sensitivity and accuracy of the combined detection of multiple TAABs were higher (83.03% and 78.33%), respectively, which was significantly better than the results of the present study, which might be mainly related to the higher number and different types of selected antibodies in Xiao et al.’s study. The results were reanalyzed with the remaining five antibodies by eliminating the two antibodies with low positive expression rates, P53 and GAGE7, and it was found that their sensitivity was 46.22%, specificity was 93.86%, and accuracy was 69.53%, which was comparable to that of the 7-TAAB combination assay. Therefore, a new set of antibody profiles specifically for ESCC should be developed in the future; this would not only reduce the cost of detection, but would also improve diagnostic efficacy.

Several studies (23–27) have shown that TAAB is correlated with the clinical features of some tumors, including lung cancer, endometrial cancer, thymic tumors, and ESCC, which reflect the biological characteristics of these tumors to some extent. By analyzing the expression of the seven antibodies and their clinical characteristics, we found that antibody expression was significantly correlated with some clinical characteristics of tumors. Moreover, through multivariate analysis, we found that lymph node staging and age were independent factors influencing the positive expression of ESCC antibodies, indicating that antibody expression tends to occur in patients with poor lymph node staging and advanced age. First, poor lymph node staging is often indicative of tumor progression, and second, lymph nodes themselves are immune sites; therefore, these patients may be more susceptible to immune stimulation and tend to produce antibodies more easily. Currently, there are no studies on the occurrence of TAAB in elderly patients. We speculate that this may be due to the different body conditions of patients of different ages, or perhaps the slow metabolism of elderly patients leads to the deposition of more antibodies that are easily detected, although the specific mechanism of production remains unclear. However, antibody detection is often necessary in elderly patients. We conclude that 7-TAAB expression is correlated with the clinical features of ESCC, suggesting that tumor antibody expression may be linked to the development of ESCC, which may provide a guiding basis for the assessment of ESCC in the future.

Therefore, 7-TAAB is valuable in the auxiliary diagnosis and clinical assessment of ESCC and is worthy of further study. It is hoped that more clinical studies can be conducted in the future to identify a set of new antibody profiles with higher sensitivity and specificity for ESCC and to improve the diagnostic and therapeutic dilemmas and prognosis of ESCC in China.

## 5 Conclusion

The multi-tumor-associated autoantibody combination test not only has a good auxiliary diagnostic value, but is also closely correlated with the clinical features of ESCC.

## Data Availability

The original contributions presented in the study are included in the article/supplementary material. Further inquiries can be directed to the corresponding authors.
